# The relationship between grain boundary structure, defect mobility, and grain boundary sink efficiency

**DOI:** 10.1038/srep09095

**Published:** 2015-03-13

**Authors:** Blas Pedro Uberuaga, Louis J. Vernon, Enrique Martinez, Arthur F. Voter

**Affiliations:** 1 Materials Science and Technology Division, Los Alamos National Laboratory, Los Alamos, NM, 87545 USA; 2 Computer, Computational and Statistical Sciences Division, Los Alamos National Laboratory, Los Alamos, NM, 87545 USA; 3 Theoretical Division, Los Alamos National Laboratory, Los Alamos, NM, 87545 USA

## Abstract

Nanocrystalline materials have received great attention due to their potential for improved functionality and have been proposed for extreme environments where the interfaces are expected to promote radiation tolerance. However, the precise role of the interfaces in modifying defect behavior is unclear. Using long-time simulations methods, we determine the mobility of defects and defect clusters at grain boundaries in Cu. We find that mobilities vary significantly with boundary structure and cluster size, with larger clusters exhibiting reduced mobility, and that interface sink efficiency depends on the kinetics of defects within the interface via the in-boundary annihilation rate of defects. Thus, sink efficiency is a strong function of defect mobility, which depends on boundary structure, a property that evolves with time. Further, defect mobility at boundaries can be slower than in the bulk, which has general implications for the properties of polycrystalline materials. Finally, we correlate defect energetics with the volumes of atomic sites at the boundary.

Mass transport is a key materials property, responsible for advanced functionality in different applications such as fast ion conductors^1^ and diffusion barriers^2^. The defect kinetics responsible for mass transport are also central to understanding phenomena such as radiation damage evolution^3^ and sintering^4^. It has been long established that grain boundaries, and interfaces more generally, significantly influence mass transport^5^. Thus, nanoscale architectures often exhibit enhanced diffusion compared to larger scale counterparts^6^. The importance of boundaries for influencing mass transport has led to a number of theoretical studies of defect mobility at and near grain boundaries^7^. These studies have found that defect mobility at interfaces is complex, with the boundaries exhibiting trap sites that in some cases impede the mobility of defects as compared to far from the grain boundary (GB)^8^. However, in spite of this large body of work, there are still important questions that remain unanswered, particularly relating to the interplay between grain boundaries and defects produced in extreme environments. In such conditions, the defect content at the boundaries can be significantly higher than at equilibrium such that defects begin to interact and cluster. How this clustering is influenced by GB structure and subsequently influences the overall response of the material is a crucial open question.

The use of atomistic simulations to understand the interaction of defects with GBs has a long history, begining with the pioneering work of Ingle et al. who used potentials to study vacancy interactions with twin boundaries in Fe, Mo, and W^9^. Studies of the mobility of defects at GBs began with the work of Balluffi and co-workers, who used molecular dynamics to examine vacancy migration in a Σ5 tilt boundary in Fe^10^,^11^. More recently, a number of groups have looked at a variety of important aspects of defect-boundary interactions, leveraging the computing power now available for atomistic simulations. For example, Adams and coworkers examined defect mobility at a number of twist grain boundaries in Cu, finding significant sensitivity with twist angle^12^,^13^. Sørensen et al. calculated the kinetic properties of interstitials and vacancies at two Σ5 GBs in Cu, finding that whether the vacancy or interstitial migration mechanism dominated depended on the boundary structure^14^. Tschopp et al. have calculated the interaction of interstitials and vacancies with about 170 different grain boundaries in Fe, identifying trends in interaction parameters with GB structure^15^. Most recently, Frolov et al. have examined the structure of boundaries as a function of defect content, finding that boundaries can exhibit a number of structures as a function of defect concentration, temperature, and doping concentrations^16^. They speculate that such changes in boundary structure will have an impact on radiation damage evolution, a conclusion that agrees with our previous results on the structure and subsequent energetics of defects near damaged boundaries^17^,^18^. In that case, we found that damaged GBs, modeled as boundaries with excess defect content, would have stronger interactions with residual defects in the bulk of the material that are dramatically different than their pristine counterparts. This led us to conclude that the sink efficiencies of interfaces will not be a static quantity but will evolve in a complex manner during irradiation as the steady state concentration of defects within the GB will depend on both boundary character and irradiation conditions^19^. Other properties also depend on this defect content, such as the mobility of the boundary as well as the direction of coupled motion^20^. How the defect structure at GBs evolves under driven conditions, such as irradiation, where a supersaturation of defects is created, is governed by the mobility of those defects both in the bulk as well as within the grain boundary plane. Our goal in this work is to examine the mobility of defects and defect clusters at the interface, with a focus on self-interstitials, to provide insight into this evolution. We show that one important factor that governs how interfaces influence defect evolution is the mobility of defects within the interfacial plane, which in turn depends on the structure of the interface. This demonstrates a direct correlation between interface structure, defect mobility within the interface, and sink efficiency of the interface.

## Results

### Interstitial cluster structure

**Figure 1:**
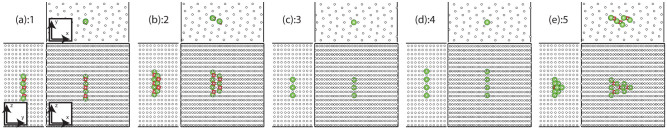
Structures of interstitial clusters within the Σ11 symmetric tilt GB for cluster sizes ranging from (a) 1 to (e) 5 interstitials. The color scheme is large (green) spheres for interstitials and (red) squares for apparent vacancies, where defects are defined using the refence lattice method^25^,^26^ with a cutoff of 0.8Å. In this scheme, the “vacancies” indicate atoms in the original GB structure that were displaced significantly upon introduction of interstitials; in each case, the net number of defects (interstitials minus vacancies) is equal to the number of extra atoms inserted into the GB (see Supplementary Information for details). The rest of the atoms, indicated by the small (white) spheres, are in their position within the undefective GB structure. The orientation of each view is given as insets in the first frame, to be compared with the orientation of the grains given in Table S1.

**Figure 2:**
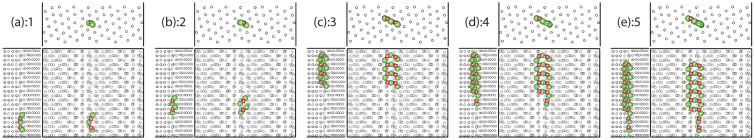
Structures of interstitial clusters within the Σ11 asymmetric tilt GB for cluster sizes ranging from (a) 1 to (e) 5 interstitials. The color scheme and the orientation of each view are the same as in Fig. 1.

**Figure 3:**
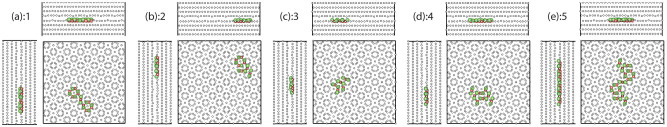
Structures of interstitial clusters within the Σ5 twist GB for cluster sizes ranging from (a) 1 to (e) 5 interstitials. The color scheme and the orientation of each view are the same as in Fig. 1.

**Figure 4:**
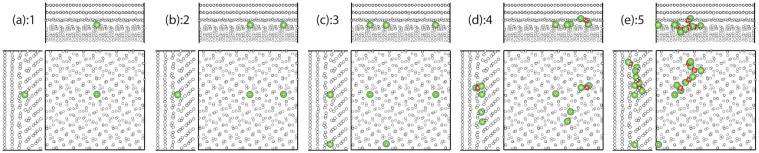
Structures of interstitial clusters within the Σ45 asymmetric tilt + twist GB for cluster sizes ranging from (a) 1 to (e) 5 interstitials. The color scheme and the orientation of each view are the same as in Fig. 1.

Figures 1,2,3,4 show the ground state structure of interstitial clusters as a function of cluster size in the Σ11 symmetric tilt, Σ11 asymmetric tilt, Σ5 twist, and Σ45 asymmetric tilt plus twist GBs, respectively, as found via a combination of adaptive kinetic Monte Carlo (AKMC)^21^ and accelerated molecular dynamics (AMD)^22^ simulations. These boundaries were chosen to represent a variety of types of GBs, from simple symmetric tilt boundaries to more general boundaries, that demonstrate a broad range of properties so that we can generalize our results to the multitude of boundaries observed experimentally. The full orientation relationships are given in Table S1 of the Supplementary Information; see Ref. 18 for details. Figures 1a, 2a, 3a, and 4a illustrate the structure of a single interstitial at each of the four boundaries. There is a clear difference between the preferred structure of the interstitial at the two pure tilt boundaries as compared with the other two GBs. In the two tilt GBs, the mono-interstitial delocalizes along the tilt axis, essentially forming a crowdion structure. While crowdion structures are common for the interstitial in bulk metals, it is usually a preferred structure in BCC metals rather than in FCC metals^23^. In Cu, the ground state structure of the interstitial is a [100] dumbbell^24^. Thus, the tilt axis breaks the symmetry of the material and lowers the energy of the crowdion structure. This has important consequences for the mobility of these structures, as we will discuss below. In contrast, for the two GBs with twist character (Σ5 and Σ45) the interstitial is much more localized. In the Σ5 boundary, the mono-interstitial forms a split interstitial structure, though the neighboring rings of the boundary structure rotate to accommodate the interstitial. In the Σ45 boundary, the interstitial resides in a pure interstice formed at the GB, with little displacement of the surrounding Cu atoms.

As the size of the interstitial cluster increases, the structural motif of these clusters in the two tilt boundaries start to diverge. In the Σ11 symmetric GB, once the cluster reaches a size of 3 interstitial atoms (Fig. 1c), it becomes more localized, losing the crowdion character it had at sizes 1 and 2. In the Σ11 asymmetric GB, on the other hand, the cluster retains its extended structure for all sizes considered, such that at the largest size we examined, containing 5 interstitial atoms, the cluster nearly extends through the length of our periodic cell (Fig. 2e). For the Σ5 GB, as the interstitial cluster size is increased, the rotations of the rings start to disappear to the point that, by sizes 3 and 4, they are no longer part of the structure (Fig. 3c–d). However, they reappear for the cluster containing 5 interstitials, which can be viewed as an agglomeration of 2 + 1 + 2 clusters, connected by the rotated rings (Fig. 3e). The behavior for the Σ45 GB is significantly different from the others. As mentioned above, there is a preferred interstice for the interstitial in this structure. For the simulation cell we constructed, there are six equivalent interstice sites within the GB plane. These sites are strongly preferred by the interstitial such that the addition of new interstitials results in the filling of these sites before any significant clustering occurs. That is, the interstitials remain more or less independent, interacting only weakly. As the cluster size increases, there does appear to be some weak clustering, as indicated by the reconstructions apparent in Figs. 4d–e, but, as we discuss next, the tendency for interstitials to cluster within the Σ45 boundary is certainly weak.

**Figure 5:**
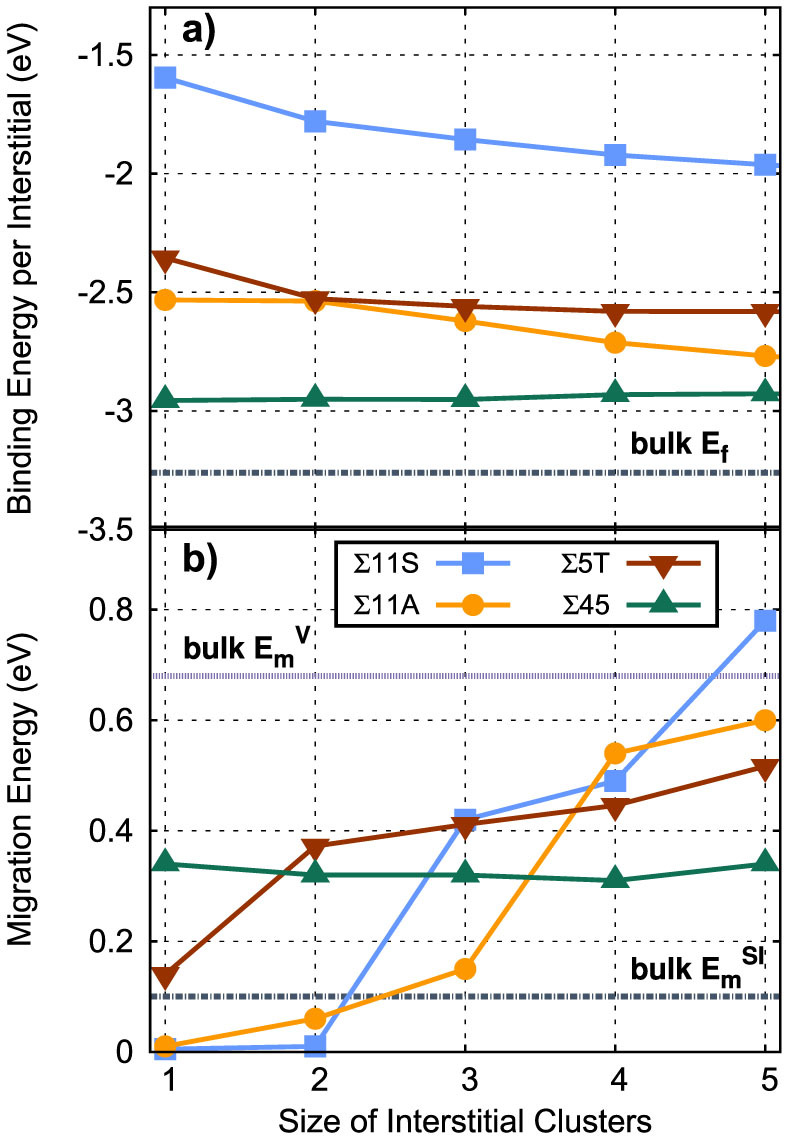
(a) Binding energy and (b) migration energy of interstitial clusters in the four GBs as a function of size. In (a), the formation energy of an interstitial in bulk Cu is indicated, as this is a lower bound of the binding energy of interstitials to any boundary (if the binding energy were lower, the interstitials would form spontaneously at the boundary). In (b), the migration energies of interstitials and vacancies in bulk Cu are indicated for comparison.

Figure 5a shows the binding energy of these interstitial clusters as a function of cluster size in each of the four boundaries. Here, the binding energy is defined as the energy of the cluster within the boundary relative to isolated interstitials in bulk Cu: 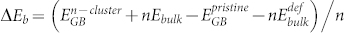, where Δ*E_b_* is the binding energy of the cluster normalized by the number of defects in the cluster, 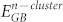 is the energy of a cluster of size *n* at the GB, 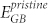 is the energy of the pristine GB, *E_bulk_* is the energy of bulk Cu, and 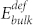 is the energy of the point defect in bulk Cu. Thus, a negative binding energy means there is a net binding of the cluster to the GB. Further, a decrease in binding energy with cluster size means that the larger cluster is more strongly bound to the boundary than the smaller cluster, or that there is a binding of interstitials within the GB plane relative to isolated interstitials in the GB. The binding of mono-interstitials at these four boundaries as compared to placing the interstitial in bulk Cu, as we have shown previously^18^, becomes stronger (more negative) as the complexity of the GB increases such that, for the two asymmetric interfaces, the formation energy of interstitials is only marginally higher than zero. As the size of the defect clusters is increased, the clusters tend to be more strongly (a more negative binding energy) bound to the interface, indicating that there is an in-boundary binding of the interstitials. The exception is for the Σ45 GB. In this case, because the interstitials tend to reside in spatially separated trap sites within the GB, their interaction is weak and the binding energy is a constant versus the number of interstitials placed in the boundary plane, indicating no in-boundary binding of the interstitials. Of course, in all cases, as the number of interstitials is increased, new GB structures may form, as discussed by Frolov et al.^16^. The trends we see in both structure and binding energy for clusters of sizes 1–5 extend to larger clusters, as discussed in the Supplementary Information and shown in Figures S1 and S2.

### Interstitial cluster mobility

We now turn to the migration characteristics of these clusters. Figure S3 (Supplementary Information) shows the migration pathway for mono-interstitials in each of the GB structures while the energy profiles of the minimum energy paths (MEPs) for that migration are shown in Fig. S4. The resulting migration energies for clusters of sizes 1–5 are shown in Fig. 5b. These pathways and migration energies represent a full center-of-mass translation of the cluster as a complete entity, leading to net migration of the cluster. For the mono-interstitials, we find that migration in the tilt GBs is very fast, with barriers on the order of 0.01 eV or less, consistent with the fact that the structure of these interstitials is a delocalized crowdion. This migration is, naturally, along the tilt axis of the boundary. In contrast, migration of interstitials in the two GBs with twist character is significantly slower. In fact, migration of mono-interstitials within these two GBs is slower than in bulk Cu, with migration energies of 0.14 and 0.34 eV in the Σ5 and Σ45 GBs, respectively. Thus, there is an interesting contrast in behavior for mono-interstitials, with faster migration in some boundaries and slower diffusion in others.

Figure 5b reveals that this behavior is significantly changed as the size of the interstitial clusters increases. For the Σ11 symmetric GB, as the size of the clusters increases and the structures of the clusters correspondingly become more localized, the barrier for migration increases significantly, such that, by a size of 3 interstitials, the barrier to migrate is significantly higher than the corresponding barrier of a single interstitial in bulk Cu. Once a size of 5 is reached, the barrier for migration of the cluster is higher than that of a Cu vacancy diffusing in the bulk. Thus, as the size of the clusters increases, they become more and more sluggish to the point that they are significantly slower than migration in the bulk. This means that interstitials trapped in the GB in the form of clusters will remain static on the time scale of point defect arrival from the bulk to the boundary.

At the Σ11 asymmetric tilt GB, we observe similar behavior for the migration barrier of clusters versus size (Fig. 5b), but the reasons are different. As opposed to the Σ11 symmetric GB, the interstitial clusters in the asymmetric GB retain a delocalized and extended structure even for larger cluster sizes. For these clusters, the migration event itself exhibits complex concerted motions that correlate with the higher barrier. For example, the migration event of the five interstitial cluster (not shown) is rather complex, with several atoms moving in directions perpendicular to the tilt axis, which is the direction of net migration. However, the consequence is that the dependence of migration barriers for clusters versus size in this GB are qualitatively similar to those in the symmetric GB: by a size of 3 their mobility is slower than interstitials in the bulk and by a size of 5 they approach the mobility of vacancies in the bulk.

As with the two tilt GBs, the migration energies of interstitial clusters within the Σ5 twist GB increase with cluster size. For the mono-interstitial, we find a barrier of 0.14 eV, consistent with previous studies of interstitials at twist boundaries in Cu^13^. As the clusters grow in size, they tend to lose the ring structure characteristic of the mono-interstitial and the core structure, the structure minus any ring structure, spans more of the twist elements within the GB, resulting in more complex motion. The cluster of size 5 is an exception, which can be viewed as two 2 interstitial clusters joined by a split interstitial in the center.

The behavior of interstitial clusters within the Σ45 GB is an interesting exception to that observed in the previous three GBs. As described above, the interstitials tend not to cluster in this GB, preferring to reside in very specific sites within the boundary plane. This has important consequences for the migration of interstitials. For a single interstitial, the migration path takes it from one of these sites to another, as shown in Fig. S3(g–h). This pathway is rather complex, with several intermediate minima involved. Because the interstitials do not cluster, or only do so weakly, the migration barriers are essentially independent of “cluster” size. That is, the interstitials always act independently of one another. The migration energy for a single interstitial within this GB is 0.34 eV, significantly larger than the barrier for bulk migration of interstitials. This suggests that, as the structural complexity of GBs increases and trap sites exist for defects, the mobility at GBs is slower than in the bulk. We discuss the implications of this below. However, as the interstitials do not bind to each other within the boundary, this mobility remains constant with cluster size and interstitials at this GB are always faster than vacancies in the bulk.

It is interesting to contrast the behavior of mono-interstitials at these four GBs with that of vacancies. We have determined the migration barrier for mono-vacancies at each of the GBs using the same procedure outlined above for interstitials. We find that the barriers are 0.24 eV, 0.41 eV, 0.10 eV, and 0.12 eV for the Σ11 symmetric, Σ11 asymmetric, Σ5 twist, and Σ45 GBs, respectively. (The migration energy for a vacancy in bulk Cu is 0.68 eV.) Interestingly, for the GBs where mono-interstitial diffusion is fast, mono-vacancies are the slowest. Conversely, the vacancies are the fastest for the GBs in which the interstitials were the slowest. These results suggest that there is a disparity between fast interstitial and fast vacancy migration within these GBs, at least for single point defects, and, to some degree, these migration energies are anti-correlated.

### Relationship Between Defect Properties and Boundary Structure

Having determined the thermodynamic, kinetic, and structural properties of defects in a selection of GBs, here we correlate those properties with the structure of the GBs themselves. We use the volumes of atomic sites at each boundary, as determined by Voronoi construction^27^, as the structural feature representative of the boundary structure. These volumes are related to the local hydrostatic pressure, and provide a convenient measure of the local atomic structure of the boundary. For any given boundary structure, there will be a unique distribution of atomic volumes, dictated by the local atomic structure of the boundary. Of course, in systems with more than one element, other factors such as local chemistry may also play a role^28^. However, as we show below, the atomic volumes correlate with the properties of the defects within each GB in pure Cu.

**Figure 6:**
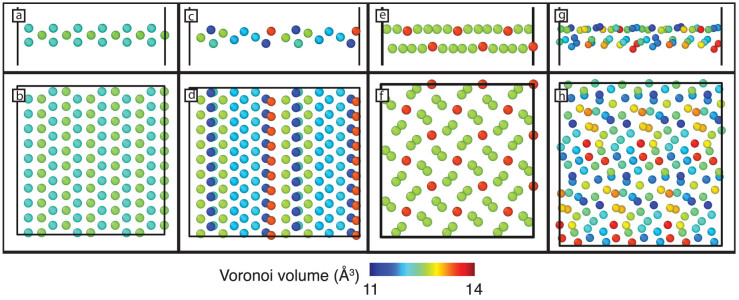
Atomic volume, relative to a site in bulk Cu (which has a volume of 11.8063 Å^3^), assocated with atomic sites at the (a–b) Σ11 symmetric tilt, (c–d) Σ11 asymmetric tilt, (e–f) Σ5 twist, and (g–h) Σ45 GBs. As indicated in the scale bar, red corresponds to larger atomic volumes and blue to lower atomic volumes. Panels a, c, e, and g are side views of the boundary plane while b, d, f, and h are top views.

Figure 6 shows the volume associated with each atomic site within each of the GB structures considered here. For the two tilt GBs, the sites with larger atomic volume are aligned in rows along the tilt axis, though the Σ11 asymmetric GB has significantly more volume in those rows than the symmetric GB. This is a close-packed <110> direction, which stabilizes the crowdion structure of the interstitial in this particular geometry. In both the Σ5 twist and the Σ45 GBs, the sites of largest atomic volume are more distributed and physically separated than in the two tilt GBs. This is particularly true for the Σ45 GB, which exhibits 6 sites of large atomic volume, corresponding to the 6 binding sites for interstitials discussed above.

**Figure 7:**
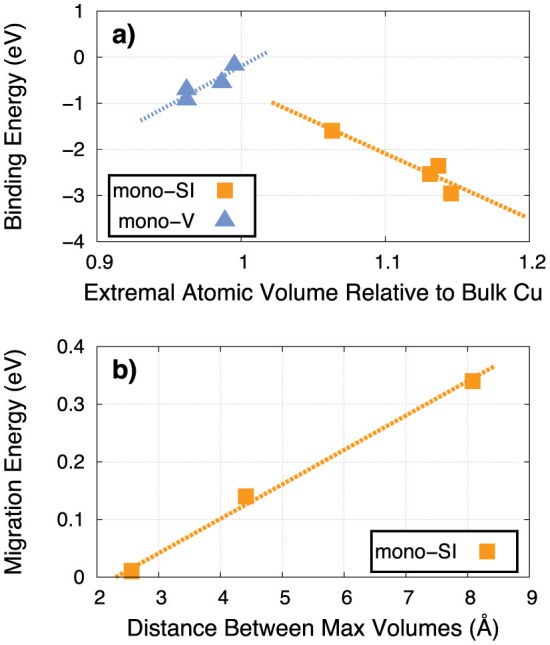
(a) Correlation between the segregation energy of vacancies with the minimum atomic volume site and interstitials with the maximum atomic volume site, relative to the atomic volume of a site in bulk Cu, at the four GBs considered. (b) Correlation between the migration barrier of mono-interstitials and the distance between maximum atomic volume sites in the four GBs (the points for the two tilt GBs overlap at the shortest distance).

The various properties of the defects correlate well with the magnitude and distribution of the atomic volumes within each GB. For example, Figure 7a compares the segregation energy of both mono-interstitials and mono-vacancies with the largest and smallest atomic volume site, respectively, at each GB. In both cases, the correlation is strong. These results indicate that interstitials are attracted to those sites with the largest atomic volume within the GB and that the strength of the attraction is directly proportional to the magnitude of the atomic volumes. The converse is true of vacancies: they are attracted to sites of minimal atomic volume.

More surprisingly, the distribution of the atomic volume within the boundary determines the migration energy. Figure 7b shows the migration energy of mono-interstitials within each GB as a function of the distance between sites with the largest atomic volume. In the two tilt GBs, this distance is minimal, lying in rows along the tilt axis, and the barrier for interstitial migration is lowest at these GBs. As the atomic volume becomes more distributed, the migration barriers increase. In fact, amongst these four GBs, the relationship between migration energy and atomic volume separation is nearly linear. Further, the distribution of atomic volumes explains the pathways for mono-interstitial migration in each boundary. In the case of the two tilt GBs, the pathways involve small shifts of atoms along the tilt axis (Fig. S3a–d). In the case of the twist GB, however, the interstitial crosses the mirror plane of the boundary, which lies between two atomic planes, each of which have sites of large atomic volume (Fig. S3e–f). Finally, for the Σ45 GB, the interstitial must execute a complicated set of moves to go from one maximal atomic volume site to the next, which are separated by relatively large distances. Thus, in each case, the interstitial shuffles between the high atomic volume sites and the distance between these sites determines the barrier. The fact that the boundary with more distributed site energies leads to slower defect migration is consistent with studies of disordered materials, which show that random distributions of site energies (as opposed to random barrier heights) can indeed retard diffusion^29^.

Finally, the distribution of atomic volume also explains why larger interstitial clusters become compact in the Σ11 symmetric GB but not in the Σ11 asymmetric GB. In both, the sites with largest atomic volume are arranged in rows along the tilt axis. Thus, in the case of a single interstitial, there is volume to delocalize along the tilt axis. However, as the size of the cluster increases, because the amount of atomic volume in the row is small for the symmetric GB, the interstitial cluster prefers to localize. This is not the case in the asymmetric GB, in which the atomic volume of these sites is relatively large. In this case, there is still ample volume for subsequent interstitials to spread along the tilt axis.

Together, these results indicate that the magnitude and distribution of the volume of atomic sites at the GBs is a critical parameter in determining defect properties at each GB. This is a more general analysis than considering, e.g., the structural motifs comprising the GBs, such as kites, as such an analysis is only applicable to tilt GBs.

### OKMC model of sink efficiency

**Figure 8:**
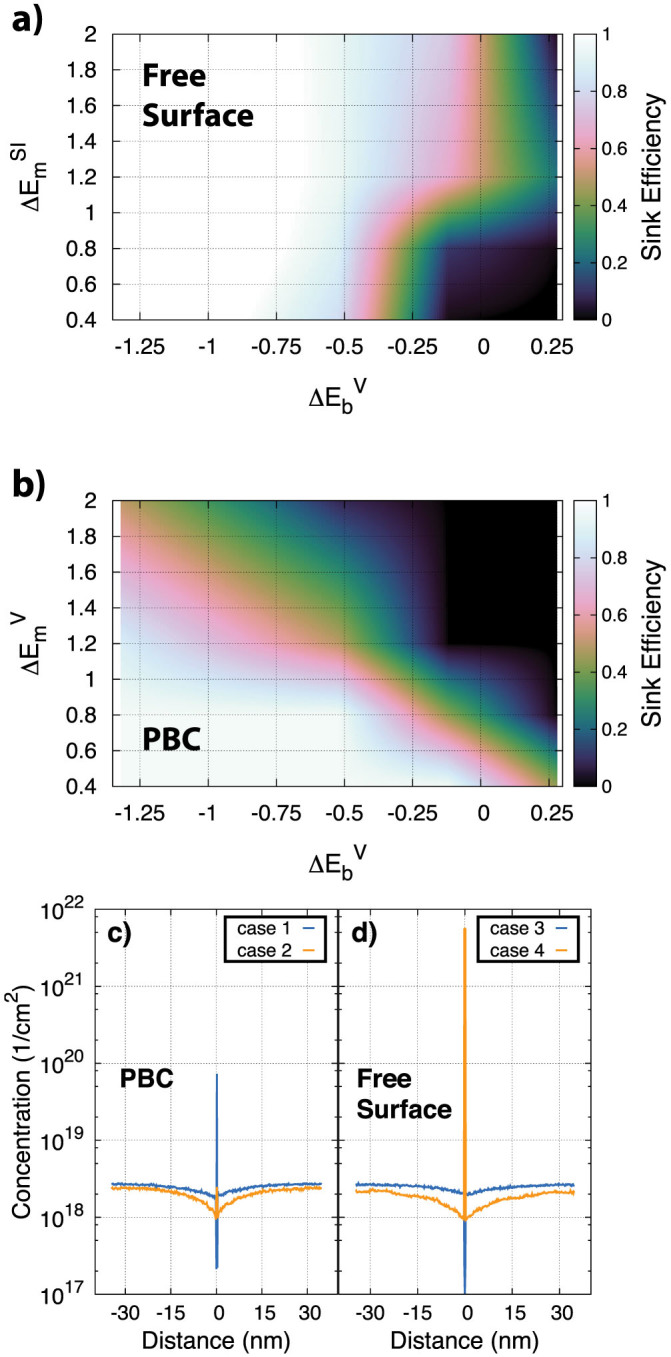
Sink efficiences as calculated using OKMC for (a) “free surface” boundary conditions and (b) periodic boundary conditions. In both (a) and (b), the sink efficiency is given as a function of the binding energy of vacancies to the GB and the migration energy of defects (interstitials in (a) and vacancies in (b)) within the GB. See text for details. (c–d) The concentration of vacancies at and near the GB for (c) the periodic model and (d) the “free surface” model. The different cases represent different defect energetics. Case 1: migration barrier for vacancy is 2.0 eV, binding energy of vacancy is 0.28 eV, mobility of interstitial is 0; Case 2: migration barrier for vacancy is 0.4 eV, binding energy of vacancy is −1.32 eV, mobility of interstitial is 0; Case 3: migration barrier for vacancy is 0.68 eV, binding energy of vacancy is 0.28 eV, migration barrier of interstitial is 2.0 eV; Case 4: migration barrier for vacancy is 0.68 eV, binding energy of vacancy is −1.32 eV, migration barrier of interstitial is 0.4 eV.

To gain insight into how these differing mobilities influence defect evolution in a nanostructured material, we constructed an object kinetic Monte Carlo (OKMC)^30^ model that incorporates the essential features of defects at these GBs. The details are in the Methods section; briefly, the model incorporates vacancy and interstitial species whose migration and binding energies within the boundary are varied to mimic the variations seen in the atomic simulations. That model is then used to calculate sink efficiencies as a function of these properties, which are provided in Fig. 8a–b. Here, the sink efficiency is defined as the flux of vacancies to the GB relative to the flux of vacancies to an ideal sink. We find that the sink efficiency of a GB is a strong function of the mobility of both vacancies and interstitials within the GB. Essentially, if in-boundary defect annihilation is reduced for any reason, the sink efficiency is also reduced. This is illustrated in Fig. 8a–b for two scenarios.

In the first case (Fig. 8a), we apply “free boundary” conditions such that interstitials within the GB plane are removed from the simulation cell with a given probability when they reach the edges of the cell (see the Methods section for details). The assumption behind this biased annihilation is that interstitials are able to escape the system by diffusing to, e.g., a triple junction or free surface far away. To ensure steady-state can be reached in the simulations, vacancies are annihilated in the bulk as well (at an arbitrary rate of 0.002/s/atom). In this set of simulations, the in-boundary migration energy of vacancies is 0.68 eV (same as in bulk Cu) while the barrier for emission of the vacancy from the boundary back into the bulk is this migration energy plus the binding energy, which is a varied parameter and represents different binding strengths of vacancies to different types of GBs. The dependency of the sink efficiency on the in-boundary interstitial migration barrier and the binding energy of the vacancy to the GB is shown in Fig. 8a. Figure 8a reveals that the sink efficiency is a strong function of the defect energetics. If interstitials are highly mobile and can escape the GB, the vacancy content at the interface begins to grow as there are fewer interstitials to recombine with those vacancies. If those vacancies are only weakly bound to the boundary (binding energies near or greater than 0 eV), they can then emit back into the grain interior, reducing the sink efficiency. In this scenario, high interstitial mobilities lead to lower sink efficiency. This is a consequence of the interstitial bias (the escape of interstitials from the system) when mobilities are high.

In the second model, the periodic model, there is no extra annihilation of defects as they reach the simulation cell boundaries. The sink efficiency as a function of in-boundary vacancy migration energy and vacancy binding to the boundary is shown in Fig. 8b. In this case, interstitials are assumed to have clustered such that their mobility is zero at the interface, mimicking a scenario in which the interstitial content at the GB is high and interstitials cannot escape the system. For these conditions, the sink efficiency is again observed to be a strong function of defect energetics. If the vacancy binding energy is modest and the vacancy migration barrier is large, the vacancy will again emit back into the bulk faster than annihilation can occur, leading to a sink efficiency less than 1. On the other hand, if the vacancy migration barrier is small, the vacancy will find an interstitial and annihilate before emission, leading to a higher sink efficiency. Thus, even in a fully periodic model without any external biases (extra annihilation terms), the sink efficiency can be very sensitive to the defect thermodynamics and kinetics within the boundary plane. In this case, high vacancy mobilities lead to higher sink efficiency as they are then more likely to find interstitials and annihilate than emit from the GB.

The conclusion from both models is that the sink efficiency of the boundary is the result of competing rates, primarily in-boundary annihilation and vacancy emission. Depending on which rates dominate, the sink efficiency will vary. Both rates depend on the accumulated defect content and the migration barriers for various types of defects within the boundary plane. These, in turn, depend on the boundary character.

Finally, not only does the sink efficiency depend on the in-boundary defect energetics, but, as illustrated in Fig. 8c–d, so does the in-boundary accumulation of vacancies. By changing the defect energetics – the migration barriers and the binding energies – the concentration of vacancies at the boundary plane vary by over three orders of magnitude. This suggests that the propensity for void nucleation at different GBs will vary as a function of the defect energetics at the GB or, rather, the GB character, which is indeed observed experimentally^31^. Further, the profiles shown in Fig. 8c–d indicate that the overall defect content at the GBs can be very complicated, with enhanced concentrations at the GBs themselves but denuded zones nearby. In the case of void formation in Cu, this would represent a high density of voids in the GB and a void denuded zone adjacent to the GBs, precisely as seen experimentally^31^. For an oxide at low temperature, this could indicate an amorphization of the boundary plane but an amorphous-free zone near the boundary.

## Discussion and Conclusions

Our results indicate that as both the defect cluster size increases and the character of GBs becomes more complex, defect mobility is reduced such that their mobility becomes slower than in bulk Cu. These results have important consequences for understanding the role of interfaces on extreme radiation environments in nanocrystalline materials. Experiments have shown that the denuded zone width in materials such as Cu varies significantly with GB character^31^. Our results suggest that an important factor is how defects migrate within the various GBs. The in-boundary mobility varies significantly with GB character and thus the sink efficiency will also vary. Further, as the defect content evolves during irradiation, the GB structure will also evolve in a way that depends on the accumulated defect concentration. Some of these structures may be extremely stable^16^ and would have their own characteristic defect mobilities. Thus, the sink efficiency of these GBs will be a complex function of both the initial GB character and the steady-state structure. The experimental results also see strong dependencies of void size and density as a function of GB character^31^, a fact that correlates with the results presented in Fig. 8c–d.

Another interesting consequence of this work is that the interaction of defects with interfaces will be very sensitive to irradiation conditions. If a material is irradiated with light ions or electrons at low dose rates, producing primarily isolated Frenkel (interstitial and vacancy) pairs, the defects will arrive at the boundary as isolated species. Particularly if the damage production rate is low, in tilt GBs they will then have time to diffuse within the boundary (as the migration rates of isolated defects within the tilt GBs is relatively fast) and ultimately will reach a far off ideal sink such as a surface^32^. In more general GBs, the ability of interstitials to escape will be limited and more vacancies will build-up at tilt GBs versus general GBs. However, under neutron or heavy ion irradiation, dense cascades are created, which cause the formation of large clusters of interstitials and vacancies, many of which have very high mobility in the bulk. These can diffuse to boundaries and essentially become trapped as their mobilities at the GBs will be significantly slower. Further, such defect clusters can be produced directly within GBs if the cascade overlaps with the boundary plane^33^. The propensity for such clustering is higher in the tilt GBs than in the more general Σ45 GB. In such cases, the tilt GBs will tend to accumulate more interstitials as the rate of interstitial arrival is much faster than interstitial removal via diffusion along the interface. Thus, there will be many more interstitials for vacancies to interact with via mechanisms such as interstitial emission^17^. This means that there is a contrast in behavior between tilt GBs and other GBs as a function of irradiation conditions. For light ion irradiation, annihilation mechanisms will be suppressed more at tilt GBs than at other GBs, while the opposite will be true under heavy ion damage.

Most probably, defect clustering and mobility within the interface plane is not the sole factor determining interface sink efficiency. Other factors are certainly important. For example, the strain fields of the boundaries themselves influence their interaction with defects and the resulting sink efficiency^34^. Grain boundaries can also significantly modify the defect production during the collision cascades themselves, which influences sink properties^33^. However, our results do indicate that defect mobility within interfaces is an important and, heretofore overlooked, factor in governing the interaction of interfaces and defects during irradiation.

Together with our previous results on defect interactions with GBs^17^,^18^, these results led to a view of defect evolution in nanocrystalline materials that is significantly more subtle and complex than previously thought. Interfaces are not ideal sinks for defects, a point that has been long recognized. However, the consequences of that fact include the conclusion that interfaces are not static entities in extreme conditions such as irradiation. They evolve over time, possibly reaching a steady state defect structure that has significantly different properties than the pristine interface. In fact, the structure can completely change as the defect content is changed at the interface^16^. Further, the steady state structure of the interface will be sensitive to the irradiation conditions as that will determine the predominant form of defect clusters at the interface and thus their mobilities and ultimately their lifetimes within the interface. Therefore, we conclude that predictions of damage evolution within a nanocrystalline material necessitate a deep understanding of how the properties of defects at GBs and interfaces depend on interfacial character and how, in turn, that same character is changed by the defect content within the interface.

Of course, boundaries and interfaces in real materials are more complicated than considered here. They contain steps^35^,^36^,^37^,^38^,^39^, disclinations^40^,^41^,^42^,^43^, impurities^44^,^45^,^46^, and point defects^16^,^18^,^47^. In fact, because the formation energy of point defects is so small at some of these boundaries, they will be present even at equilibrium^18^. These various imperfections will complicate the properties of boundaries and, in particular, will lead to changes in defect migration energies. One of the motivations of the current work is to elucidate how point defects behave in ideal boundaries as even in these simplest cases the migration behavior of point defects has not been established. Our results provide a foundation from which the effect of complicating factors can be interpretted. Further, because the OKMC model is general and is not directly mimicking the properties of any specific boundary, it, in effect, accounts in a generic sense for these types of imperfections. That is, the net effect of these imperfections will be to modify the migration and thermodynamic energetics of defects with in the boundary plane, and the OKMC model arbitrarily accounts for these changes.

Finally, our results have more general implications for transport in polycrystalline materials beyond radiation effects. It is typically assumed that defect mobility is faster at interfaces than in bulk material. Our results indicate this is not the case, with defects showing the reverse tendencies in more general boundaries. In the cases we have examined, defect mobility is only faster than in the bulk at the pure tilt GBs, along the tilt axis. Thus, there is a subset of all possible boundaries in which defect mobility is faster than in the bulk, and another set where it is slower. Further, the size of the second set of boundaries is relatively larger for defect clusters. At this point, we cannot say how large each set is. However, our results suggest that the enhanced diffusion often observed in experimental studies of polycrystalline materials is at least in part governed by higher defect concentrations at interfaces, where their formation energy is much lower. Therefore, even if the mobility per defect is slower, the overall diffusion constant could still be faster. These conclusions are similar to those reached in Ref. 13. In addition, as the formation energy of interstitials is often particularly low at interfaces^18^, the concentration of interstitials might be high enough to cluster, reducing the in-boundary mobility further, even in equilibrium conditions where only thermal populations of defects exist. We have not explicitly considered the equilibrium defect behavior as, under irradiation, the irradiation-induced defect content is typically orders of magnitude greater than the equilibrium concentration. However, our results lead us to suggest that the higher diffusivities observed in polycrystalline materials is not a consequence of higher mobilities at interfaces, but rather higher defect concentrations. Our results thus provide new insight into mass transport at polycrystalline materials beyond irradiation conditions.

## Methods

Here, we use a combination of accelerated molecular dynamics (AMD)^22^, adaptive kinetic Monte Carlo (AKMC)^21^, and object kinetic Monte Carlo (OKMC) to determine the mobility of defects at GBs and the impact on GB sink efficiency. In particular, using AMD and AKMC, we examine how the GB structure changes both the mobility of isolated point defects and the behavior of defect clusters, focusing on the mobility of interstitial clusters as a function of cluster size at four representative GBs. We then use OKMC simulations to probe the influence of the atomistic results on the sink efficiency of GBs.

We use Cu as a model system to simulate defect migration at GBs, using an embedded atom method (EAM) potential^48^ as developed by Mishin et al.^24^. We focus on the same four boundary structures that we used to study defect absorption; the structures of the boundaries and the simulation sizes are detailed in Ref. 18. These GBs are expected to represent a range of GB structures observed in real materials. We then use AMD and AKMC simulations to anneal defect structures and identify migration pathways of the lowest energy structures. We place a given number of defects at the GB, typically starting from the lowest energy structure of the next smaller defect cluster and adding one more defect within the GB plane, and annealing the structure. Here, we use a modified version of the AKMC algorithm in which the acceptance of an event is based not on the rate but on a Metropolis probability defined by the difference in energy between the initial and final states, to accelerate the time to find low energy structures, similar to the approach of Mousseau and Barkema^49^. We identify the lowest energy structure of the new defect size found during the course of these simulations and then use this structure in standard AKMC simulations to find the migration pathway. Typically, especially for larger clusters in the more complex boundary structures, the pathways for net diffusion involve multiple intermediate minima. Temperature accelerated dynamics^50^, which uses a molecular dynamics trajectory to find events, was used to find the pathways for smaller clusters that exhibited low migration energies as the negative curvatures associated with these paths and which the AKMC simulations rely upon were very soft and lead to convergence issues with the AKMC saddle searches.

An OKMC^30^ model was developed that captures the salient physics from the atomistic simulations. In this model, the defects are treated as abstract objects whose energetics (thermodynamic interactions with the GB and migration energies at and near the GB) are defined a priori. The GB itself is modeled as a plane with no explicit structure. Rather, the effective structure of the GB is accounted for via the migration and binding energies chosen. Thus, the model for any given simulation is defined by the rates of migration of interstitial and vacancy species within the GB plane and the rate of emission of vacancies back into the bulk region of the material, which is determined by the binding energy (interstitials are assumed to bind so strongly to the GBs, as per Ref. 18, that they cannot emit back into the bulk). The model does not explicitly account for clustering, but does account for changes in migration energy associated with clustering via the change in binding and migration energies. While clustering would lead to a change in behavior with time, as fast moving mono-interstitials become trapped in slow moving interstitial clusters, and this time dependence is not captured in this model, our model represents two limiting cases of this behavior and provides qualitative insight into the relationship between sink efficiency and defect properties at the grain boundary. Two sets of boundary conditions were applied: periodic boundary conditions in which interstitials were allowed to move from one side of the simulation box to the other, and “free surface” boundary conditions in which it was assumed that interstitials can escape to some far away sink. When an interstitial is at the GB and it reaches the edge of the simulation cell, it is annihilated with a probability of 0.1. The OKMC model was then used to determine sink efficiencies and in-boundary vacancy concentrations as a function of the kinetic parameters of the defects. Sink efficiencies were defined as the flux of vacancies to the GB relative to the flux of vacancies to an ideal sink. All OKMC simulations were performed at 723 K and run until steady state was reached.

## Author Contributions

B.P.U. wrote the main manuscript text. L.J.V. and B.P.U. performed the atomistic calculations. E.M. performed the OKMC calculations. A.F.V. helped interpret the results. All authors reviewed the manuscript.

## Supplementary Material

Supplementary Information Supplementary Information for The relationship between grain boundary structure, defect mobility, and grain boundary sink efficiency
