# Supplementary material for: The relationship between grain boundary structure, defect mobility, and grain boundary sink efficiency
Source: Sci Rep. 2015 Mar 13;5:9095. doi: 10.1038/srep09095 (PMC4357896; doi:10.1038/srep09095)
Supplement: Supplementary Information — for The relationship between grain boundary structure, defect mobility, and grain boundary sink efficiency [file srep09095-s1.pdf]

Supplementary Information for

The relationship between grain boundary structure, defect mobility, and grain boundary sink efficiency

Blas Pedro Uberuaga,[1] Louis J. Vernon,[1] Enrique Martinez,[1] and Arthur F. Voter[2]

[1]*Materials Science and Technology Division, Los Alamos National Laboratory, Los Alamos, NM, USA*

[2]*Theoretical Division, Los Alamos National Laboratory, Los Alamos, NM, USA*

1. Grain Boundary Orientation Relationships

The following table, adapted from Ref. 16, provides the full orientation relationships for the four grain boundaries discussed in the manuscript.

Table S1. *Orientation relationships of the four grain boundaries examined in this work. The Cartesian coordinates correspond to the labels in Figures 1-4 and S1.*

Boundary	Upper Grain			Lower Grain		
	x	*y*	*z*	*x*	*y*	*z*
Σ5 twist	[3 0 -1]	[0 1 0]	[1 0 3]	[3 0 1]	[0 1 0]	[-1 0 3]
Symmetric Σ11 tilt	[3 2 -3]	[-1 3 1]	[1 0 1]	[3 -2 -3]	[1 3 -1]	[1 0 1]
Asymmetric Σ11 tilt	[5 4 -5]	[-2 5 2]	[1 0 1]	[1 8 -1]	[-4 1 4]	[1 0 1]
Σ45	[2 1 0]	[0 0 1]	[1 -2 0]	[0 1 -2]	[2 2 1]	[5 -4 -2]

2. Structure of Interstitial Clusters and the Reference Lattice Method

In Figs. 1-4, the structures of interstitial clusters at each grain boundary are shown as determined via the reference lattice method. In this method, the position of atoms in the defective structure are compared with those in the clean structure. Thus, in this scheme, an interstitial atom is defined as any atom in the defective structure that does not have an equivalent atom in the original structure within some cutoff distances (0.8 angstroms here). Similar, a vacancy is defined as any atom in the original structure with no corresponding atom within the cutoff in the defective structure. Thus, vacancies represent atoms that have been displaced from their original position by more than the cutoff and illustrate how the inclusion of interstitials within the grain boundary plane leads to reconstruction of the original structure. In each case, however, the sum of the number of interstitials and of the number of vacancies must equal the net number of defects (in the case of Figs. 1-4, interstitials) introduced within the boundary

plane. That is, the number of green spheres minus the number of red cubes is equal to the number of atoms inserted into the boundary.

3. Ten Interstitial Clusters

In order to provide some insight into the generality of the trends versus cluster size described in Figure 5, we have performed the same AKMC type simulations for interstitial clusters containing 10 interstitials. In these cases, we were not able to systematically search for a migration pathway, but using the MMC algorithm described in the main text, we searched for low energy structures though, because of the larger space the searches for these larger clusters entail, we have less confidence that we have found the true ground state. That said, this data is provided here to extend at least qualitatively the trends discussed in the main text.

The lowest energy structures found are shown in Figure S1. The basic structural motifs discussed in the main text for cluster sizes 1-5 continue for clusters containing 10 interstitials. In the Σ11 symmetric tilt boundary, the cluster is compact, while for the Σ11 asymmetric tilt boundary, the cluster extends across the whole periodic dimension, reconstructing the entire row along the tilt axis (though it is compact in the directions normal to the tilt axis, again consistent with the free volume distribution shown in Figure 6 in the main text). This is again a consequence of the greater free volume at the asymmetric boundary. For the other two boundaries, we see that there is less of a tendency to form larger clusters. In both cases, the 10 interstitial cluster extends across two atomic planes at the boundary (Figure S1e and S1g). This behavior is reflected in the binding energy associated with these clusters (Figure S2). For the two tilt boundaries, there is an increase in binding energy (per interstitial) going to the larger 10 interstitial cluster. For the other two boundaries, this trend is not apparent, suggesting that larger clusters form only weakly if at all.

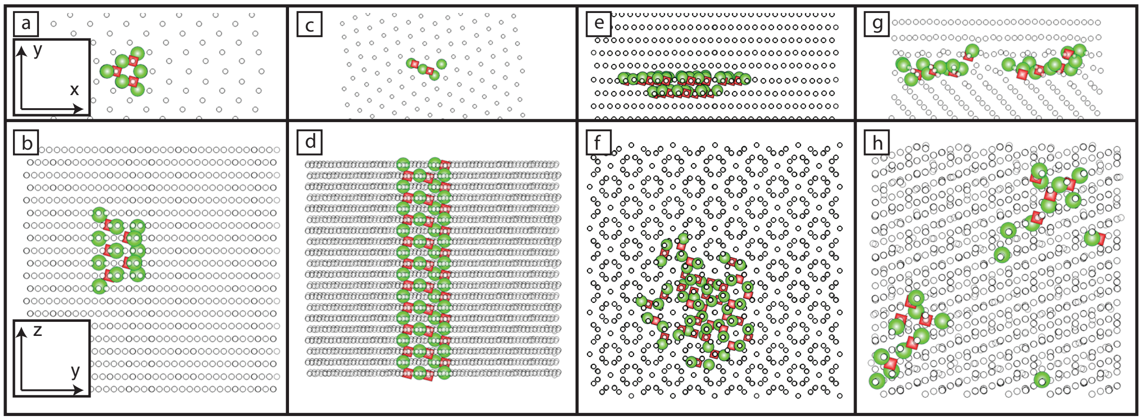

***Figure S1**. The structure of ten interstitial clusters in the (a-b) Σ11 symmetric tilt GB, (c-d) Σ11 asymmetric tilt GB, (e-f) Σ5 twist GB, and (g-h) Σ45 asymmetric tilt plus twist GB. (a,c,e,g) are side views of the cluster, looking edge-on at the GB plane and (b,d,f,h) are top views of the cluster, looking down on the GB plane*

(similar to Figures 1-4 in the main text). The color scheme is the same as in Figures 1-4 in the main text.

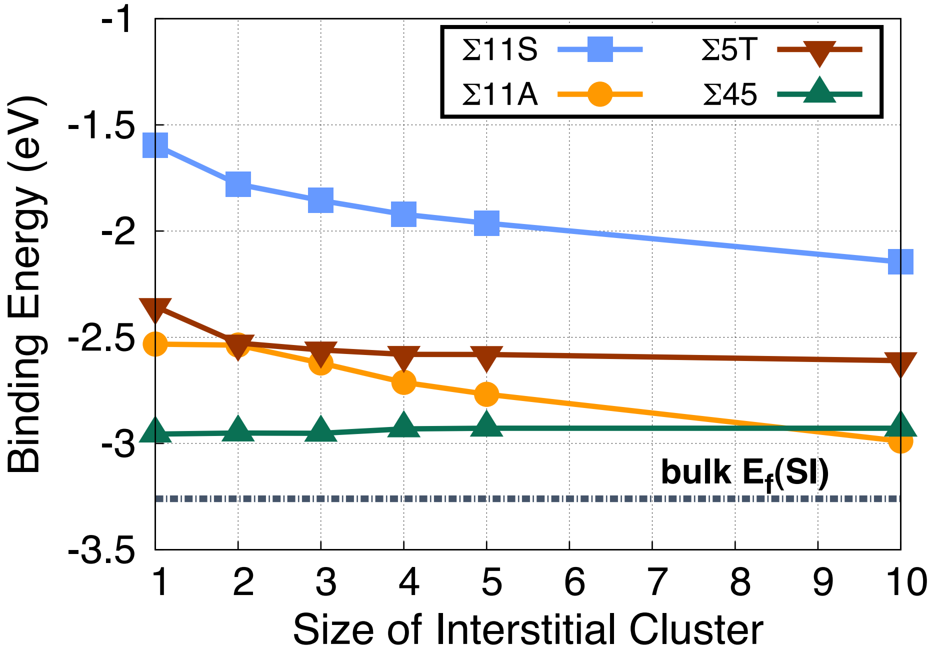

***Figure S2**. The binding energy of interstitial clusters in the four boundaries, similar to Figure 5 in the main text, but including the 10 interstitial clusters.*

4. Migration Pathways for Mono-interstitials

Figure S3 shows the migration pathways for mono-interstitials as found from AKMC and TAD simulations. These pathways represent the shortest paths in terms of number of states that take the interstitial from one lowest energy position within the grain boundary plane to an equivalent but translated position.

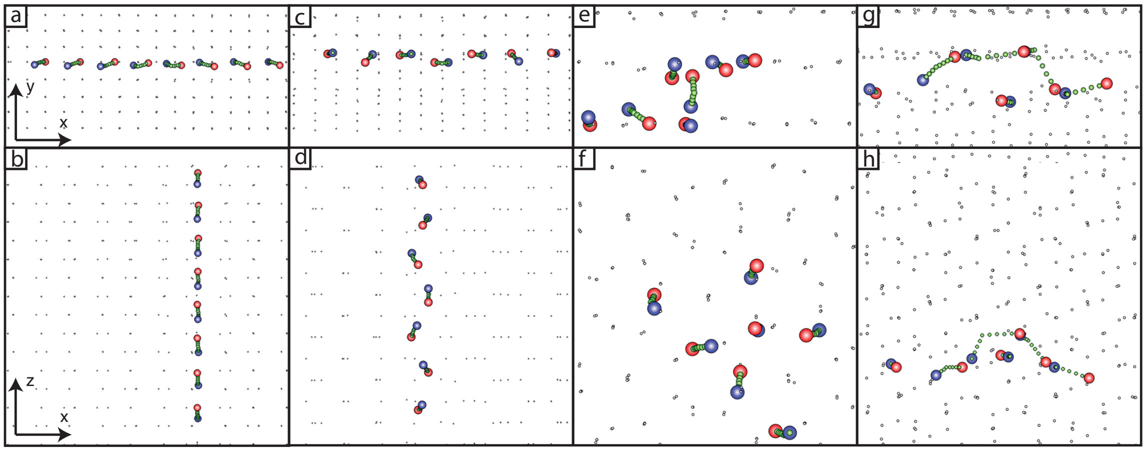

Figure S3. *The migration pathway for a mono-interstitial diffusing in the (a-b) Σ11 symmetric tilt GB, (c-d) Σ11 asymmetric tilt GB, (e-f) Σ5 twist GB, and (g-h) Σ45 asymmetric tilt plus twist GB. (a,c,e,g) are side views of the pathway, looking edge-on at the GB plane and (b,d,f,h) are top views of the pathway, looking down on the GB plane. The color scheme for the pathways is as follows: large blue spheres indicate the initial position of atoms and large red spheres the final position of atoms. Small green spheres indicate intermediate positions of the atoms, interpolated between their initial position, their saddle point, and their final position. In each figure, the atoms that moved more than x during the event are highlighted with the large spheres, where x = 0:2, 0.3, 0.4, and 0.35 Å for the Σ11 symmetric, Σ11 asymmetric, Σ5, and Σ45 boundaries, respectively (these values were chosen to best illustrate the pathway).*

Figure S4 shows the energy profiles for the pathways for the mono-interstitial at each of the four boundaries. We note that the behavior of the mono-interstitial in the Σ5 twist boundary is more complicated than illustrated in Figs. S3 and S4. The net migration of the lowest energy structure, shown in Fig. 3.1 in the main text, consists of two steps. There is first an activation step with a barrier of 0.14 eV in which the interstitial localizes (the ring structure in Fig. 3.1 untwists). This localized state has an energy of only 0.01 eV relative to the ground state. The interstitial can then diffuse through the boundary with barriers of 0.07 eV, relative to the localized state. This is the mechanism that is illustrated in Figs. S3 and S4.

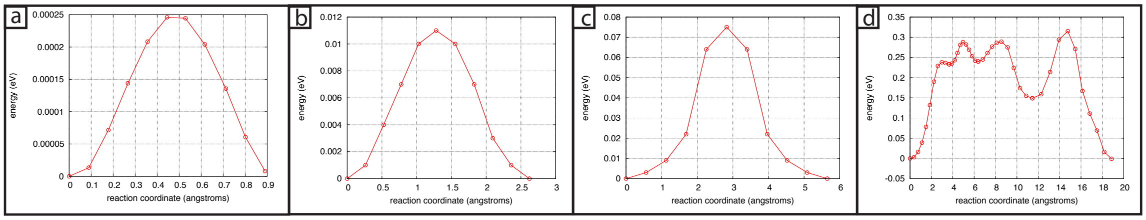

Figure S4. *Energy profiles for MEPs of the mono-interstitial pathway at the (a) Σ11 symmetric, (b) Σ11 asymmetric, (c) Σ5, and (d) Σ45 boundaries.*
